# Analyzing seasonal patterns of wildfire exposure factors in Sardinia, Italy

**DOI:** 10.1007/s10661-014-4175-x

**Published:** 2014-12-04

**Authors:** Michele Salis, Alan A. Ager, Fermin J. Alcasena, Bachisio Arca, Mark A. Finney, Grazia Pellizzaro, Donatella Spano

**Affiliations:** 1 Department of Science for Nature and Environmental Resources (DIPNET), University of Sassari, Via De Nicola 9, 07100 Sassari, Italy; 2 Euro-Mediterranean Center on Climate Change (CMCC), IAFENT Division, Via De Nicola 9, 07100 Sassari, Italy; 3 USDA Forest Service, Pacific Northwest Research Station, Western Wildland Environmental Threat Assessment Center, 3160 NE 3rd Street, Prineville, OR 97754 USA; 4 National Research Council (CNR), Institute of Biometeorology (IBIMET), Traversa La Crucca 3, 07100 Sassari, Italy; 5 Rocky Mountain Research Station, Fire Sciences Laboratory, 5775 Highway 10 West, Missoula, MT 59808 USA

**Keywords:** Burn probability, Fire exposure, Fire risk, Mediterranean areas, MTT algorithm, Seasonal patterns, Fire modeling

## Abstract

In this paper, we applied landscape scale wildfire simulation modeling to explore the spatiotemporal patterns of wildfire likelihood and intensity in the island of Sardinia (Italy). We also performed wildfire exposure analysis for selected highly valued resources on the island to identify areas characterized by high risk. We observed substantial variation in burn probability, fire size, and flame length among time periods within the fire season, which starts in early June and ends in late September. Peak burn probability and flame length were observed in late July. We found that patterns of wildfire likelihood and intensity were mainly related to spatiotemporal variation in ignition locations, fuel moisture, and wind vectors. Our modeling approach allowed consideration of historical patterns of winds, ignition locations, and live and dead fuel moisture on fire exposure factors. The methodology proposed can be useful for analyzing potential wildfire risk and effects at landscape scale, evaluating historical changes and future trends in wildfire exposure, as well as for addressing and informing fuel management and risk mitigation issues.

## Introduction

Understanding spatiotemporal patterns in wildfire exposure and risk and the underlying drivers remains a key challenge for fire scientists and land managers, especially in the case of large catastrophic fires that cause most of the human, financial, and ecological losses (Pereira et al. 2005; Ager et al. 2012; Parisien et al. 2012; Ganteaume and Jappiot 2013). While fuel moisture and load, extreme weather, and topography all contribute to wildfire risk (Pyne et al. 1996; Moreira et al. 2011), human behavior as it relates to the timing and location of ignitions is also a key factor (Martinez et al. 2009; Skinner et al. 2009; González-Olabarria et al. 2012; Lovreglio et al. 2012; San Miguel-Ayanz et al. 2013). The allocation of fire suppression resources during peak periods of fire occurrence is another major determinant in terms of ignitions escaping initial attack and becoming large fires (Hirsch and Martell 1996; Arienti et al. 2006; Plucinski et al. 2012; Plucinski 2013). In the Mediterranean region, which has seen a dramatic increase in wildfire activity in the 1990s (Moreno et al. 1998; Pausas 2004; Viegas et al. 2009), variation among fire seasons has been specifically linked to fuel moisture and load and to weather conditions (Mouillot et al. 2003; Pellizzaro et al. 2007; Pausas and Fernandez-Munoz 2012; Curt et al. 2013; Xystrakis et al. 2013). For instance, between early July and the end of August, the Mediterranean basin areas experience periods of strong winds, high temperatures, and low atmospheric relative humidity (Pereira et al. 2005; Cardil et al. 2013, 2014). Under such extreme environmental conditions, spread and intensity of wildfires often overwhelm the suppression capabilities of ground and aerial fire-fighting efforts, resulting in a higher probability of large fires and associated impacts (Arienti et al. 2006; Moreira et al. 2011; Brotons et al. 2013; Cardil and Molina 2013).

Several studies defined and characterized wildfire risk in the Mediterranean Basin using a variety of approaches. Most studies have either investigated the relationship between fire risk, meteorological conditions, and biophysical variables (Verde and Zezere 2010; Moreira et al. 2011; Karali et al. 2013) or quantified risk as ignition probability maps, coupled with statistical analyses that explained observed ignition patterns with socioeconomic factors. These latter studies employed a wide range of statistical and methodological approaches such as logistic regression, Bayesian statistics, and neural networks (Vasilakos et al. 2007; Romero-Calcerrada et al. 2008; Catry et al. 2009; Martinez et al. 2009; Ager et al. 2014b).

An alternative body of literature has employed wildfire simulation modeling to better understand spatiotemporal patterns in wildfire risk and associated drivers. These studies include large-scale exposure assessments (Thompson et al. 2011; Ager et al. 2014a), evaluation of fuel treatment strategies (Ager et al. 2010a, b), endangered species habitat protection (Ager et al. 2007), landscape-level forest planning (González-Olabarria and Pukkala 2011), watersheds protection (Thompson et al. 2013b), incident-level decision support (Calkin et al. 2011b; Noonan-Wright et al. 2011), localized assessment of risk to structures or values (Ager et al. 2012; Haas et al. 2013; Salis et al. 2013, 2014; Thompson et al. 2013a), and evaluation of climate change impacts (Arca et al. 2012). The use of wildfire simulations in fire exposure assessment at landscape scales allows the mapping of burn probability and associated fire intensities in relation to key drivers including weather, fuel, topography, and spatial ignition patterns (Miller and Ager 2013).

In this paper, we use wildfire simulation modeling to explore spatiotemporal patterns of wildfire exposure factors for Sardinia, Italy. The island is one of the regions most affected by wildfires in Italy and is a fire-prone area of particular interest also because it represents well the Mediterranean environments and conditions. In contrast to previous work in this area, we examined the combined effects of seasonal variation of weather, fuel moisture and types, topography, and historical fire occurrence on wildfire exposure. Specifically, we investigated how burn probability, fire size, and intensity change over the fire season and how the resulting seasonal pattern leads to specific trends in wildfire exposure to highly valued resources. We then discuss how the study can improve wildfire risk management on fire-prone Mediterranean landscapes.

## Methods

### Study area

**Fig. 1:**
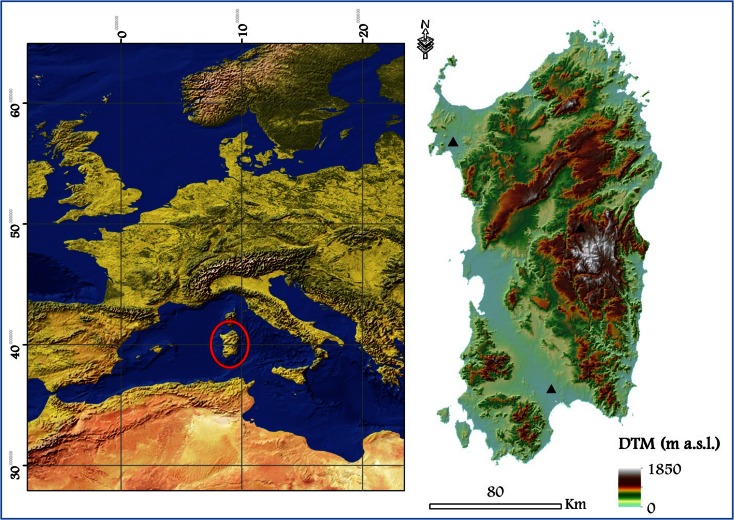
Location of the island of Sardinia, Italy, in the Mediterranean basin. The *figure on the right* shows the elevation map (as derived by the 10-m DTM—digital elevation model—of Sardinia) of the island. The weather stations used in this study are represented by the *black triangles*

**Fig. 2:**
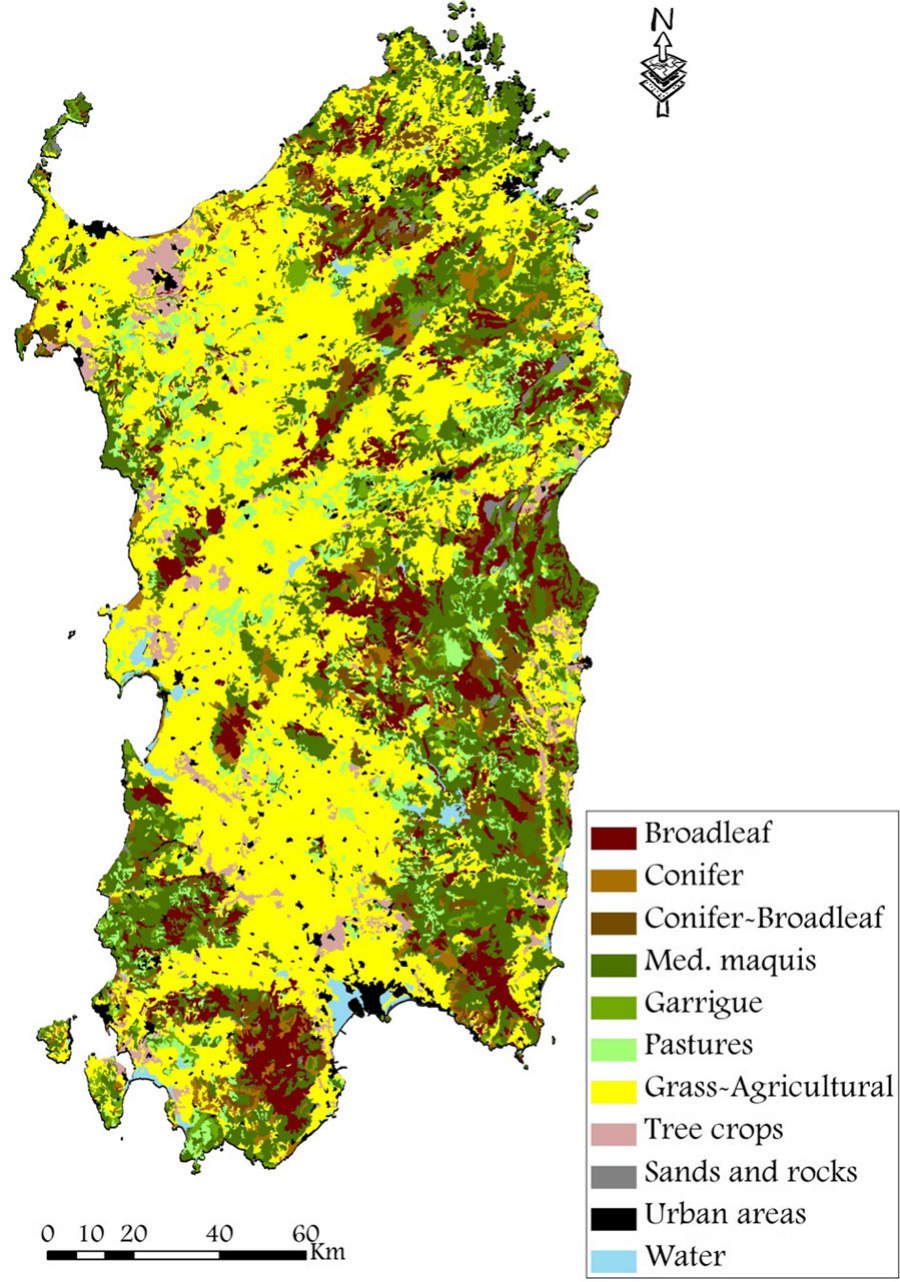
Primary vegetation and land types in Sardinia as derived from the Sardinia Land Use Map of 2003 (www.sardegnageoportale.it)

The study area is Sardinia, Italy, the second largest island of the Mediterranean Basin (Fig. 1). The island has a topography characterized by the most relevant hills and mountains on the eastern side (Fig. 1) and plains located in the western part, with two main flat areas: Campidano in the South, and Nurra in the North. The highest peak is situated in central Sardinia and reaches about 1850 m a.s.l. (Fig. 1). The climate, albeit with some gradients from the coastal areas to the mountains, and from South to North, is characterized by a mild and rainy period, from October to May, and a warmer one, from June to September, with peaks above 30 °C in several days, and with a low incidence of rainfall (Table 1). The annual cumulative precipitation ranges from 500 mm in the coastal areas to about 1200 mm in the mountain peaks. Approximately half of Sardinia is covered by broadleaf forests (about 16 %), in particular *Quercus* spp., and Mediterranean shrubs and garrigue (about 30 %) (Fig. 2) (Sardinia land use map of 2003, www.sardegnageoportale.it). Unlike other Mediterranean areas, the presence of *Pinus* spp. is limited (nearly 3 %). A significant part of the island is represented by grasslands and mixed agricultural areas (approximately 40 %), while the remainder is composed by herbaceous pastures, vineyards and orchards, and urban areas (Fig. 2).

**Table 1:** Average values of mean temperature (T, °C) and cumulative precipitation (PP, mm), as well as standard deviation, for the months of June, July, August, and September in Sardinia. The average values of maximum temperatures (TM) and minimum temperatures (Tm) are provided for first, second, and third decades of the abovementioned months.

Month	T	PP	TM1	TM2	TM3	Tm1	Tm2	Tm3
Jun	19.72 ± 2.35	22.03 ± 8.29	24.03	24.97	26.63	13.27	14.10	15.30
Jul	23.09 ± 1.91	7.23 ± 4.52	28.27	29.00	29.63	16.67	17.27	17.70
Aug	23.56 ± 2.23	16.90 ± 7.20	30.03	29.83	27.97	18.20	18.17	17.13
Sep	20.37 ± 2.55	42.60 ± 6.86	26.60	25.73	24.23	15.80	15.23	14.60

The weather stations of Alghero (north), Decimomannu (south), and Fonni (center) were used as reference. The data were gathered from the Aeronautica Militare Italiana—Servizio Meteorologico (2009)

### Input data for wildfire simulations

#### Fire occurrence database

**Fig. 3:**
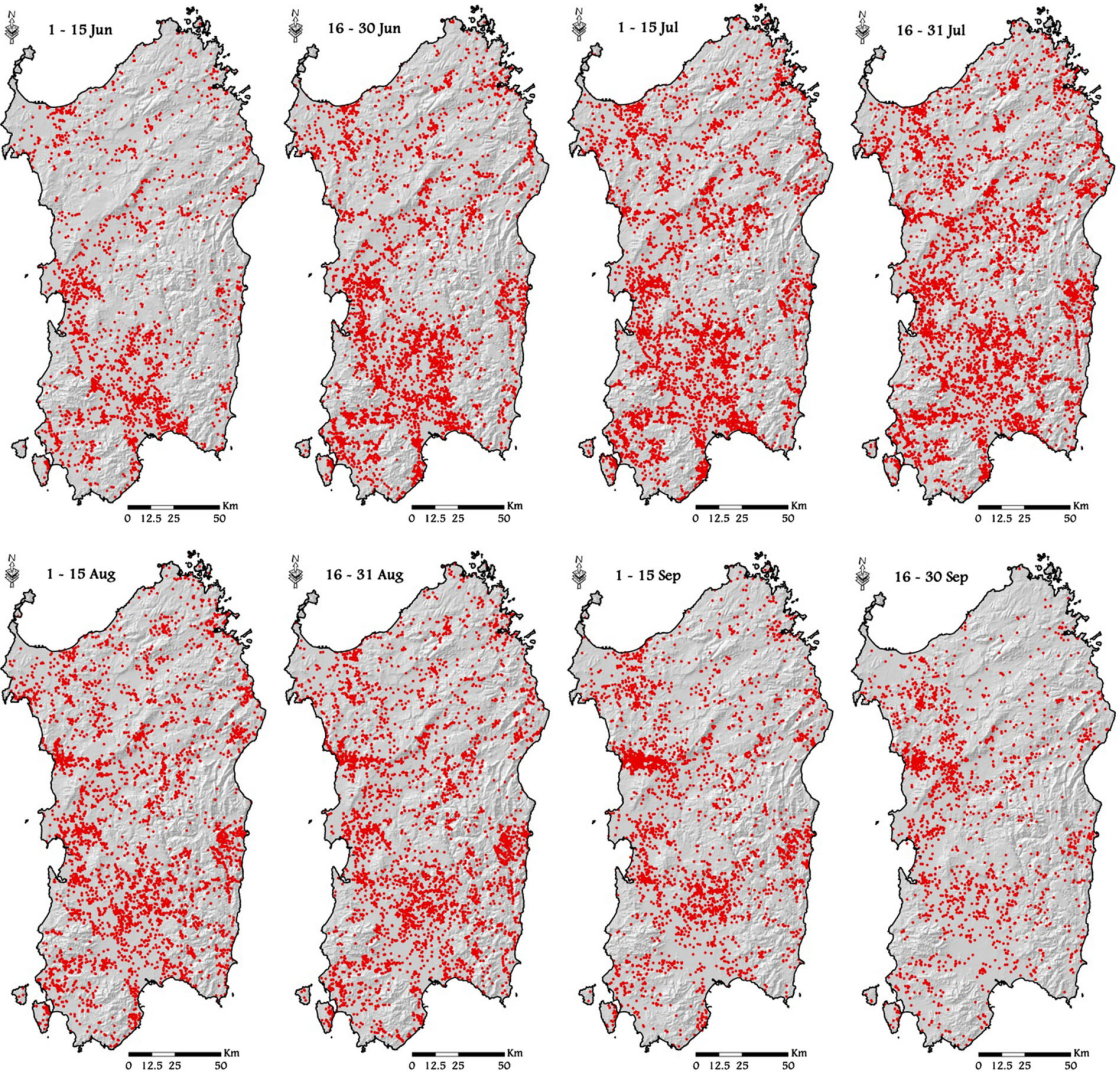
Historical fire ignition points (*red dots*) in the island, from June to September, for the study period (1995–2009). Data from the Sardinia Forest Service (personal communication, 2010)

**Fig. 4:**
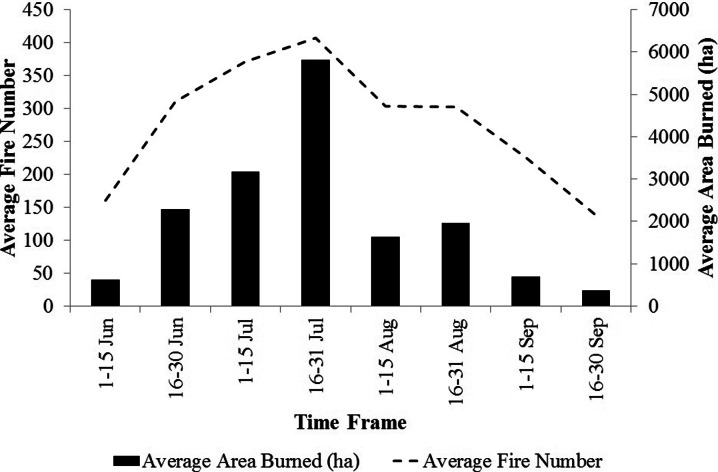
Seasonal pattern of average fire number and area burned in Sardinia, from June to September, for the study period (1995–2009). Data from the Sardinia Forest Service (personal communication, 2010)

We determined historical pattern of ignitions in Sardinia using a wildfire occurrence database that included observations from 1995 to 2009. This database consisted on information on ignition dates, municipality of ignition, coordinates, and final fire size. Within the study period (1995–2009), we analyzed all events occurred from June to September. In general, fires ignited after September and until June are relatively few in number and very small in size. We partitioned the data into eight time frames according to the ignition date as follows: 1 (1–15 June), 2 (16–30 June), 3 (1–15 July), 4 (16–31 July), 5 (1–15 August), 6 (16–31 August), 7 (1–15 September), and 8 (16–30 September) (Fig. 3). As shown in Fig. 4, Sardinia was affected by about 2,500 fires per year (∼0.104 fires km^−2^ per year), with an average annual area burned of about 20,000 ha, and peaks activity observed in the time frame 4 (16–31 July). Relatively low activity was observed in early June and late September (Fig. 4).

#### Topography and fuels

Spatial data on topography (elevation, slope, aspect) and fuels (fuel models, canopy cover) were processed with ArcFuels (Ager et al. 2011) as required by Randig (Finney 2002). The digital elevation model (10-m resolution, www.sardegnageoportale.it) was used to characterize elevation, slope, and aspect. Sardinia land use map of 2003 (shapefile, www.sardegnageoportale.it) was used as reference layer for the determination of the surface fuel models (Anderson 1982; Scott and Burgan 2005; Arca et al. 2009; Duce et al. 2012; Salis et al. 2013; Table 2). As presented in Table 2, we defined the following 12 fuel and land types: urban areas, water bodies, rocks and sands, grasslands, mixed agricultural areas, orchards and vineyards, herbaceous pastures, garrigue, Mediterranean maquis, broadleaf forests, conifer forests, and mixed forests. The canopy characteristics of the forest types were defined using the data reported by INFC (2005). Chemical and physical fuel properties were defined by associating to each fuel type either standard fuel models (NFFL (Anderson 1982) and FBM40 (Scott and Burgan 2005)) or custom fuel models specifically developed for Sardinia and Corsica vegetation (Duce et al. 2012; Salis et al. 2013) (Table 2). Both topography and fuels data were converted to ASCII files of 150-m resolution.

**Table 2:** Vegetation types derived from the Sardinian Land Use Map of 2003 (www.sardegnageoportale.it), with the relative incidence in hectares and in percentage and the respective fuel models used for the wildfire simulations

Vegetation type	Incidence (10^3^ ha)	Incidence (%)	Fuel model
Broadleaf	306.6	12.7	TL3 (Scott and Burgan 2005)
Conifer	66.5	2.8	TL6 (Scott and Burgan 2005)
Broadleaf-conifer mix	14.7	0.6	TU1 (Scott and Burgan 2005)
Mediterranean maquis	682.2	28.3	CM28 (Arca et al. 2009)
Garrigue	34.6	1.4	CM29 (Arca et al. 2009)
Pastures	162.4	6.8	CM27 (Arca et al. 2009)
Grass-agricultural lands	996.8	41.4	Mod 1 (Anderson 1982)
Tree crops	49.6	2.1	Mod 2 (Anderson 1982)
Sands and rocks	1.2	0.1	Mod 1 (Anderson 1982, fuel load reduced of 50 %)
Urban areas	66.3	2.8	NB1 (Scott and Burgan 2005)
Water bodies	26.0	1.1	NB8 (Scott and Burgan 2005)

**Fig. 5:**
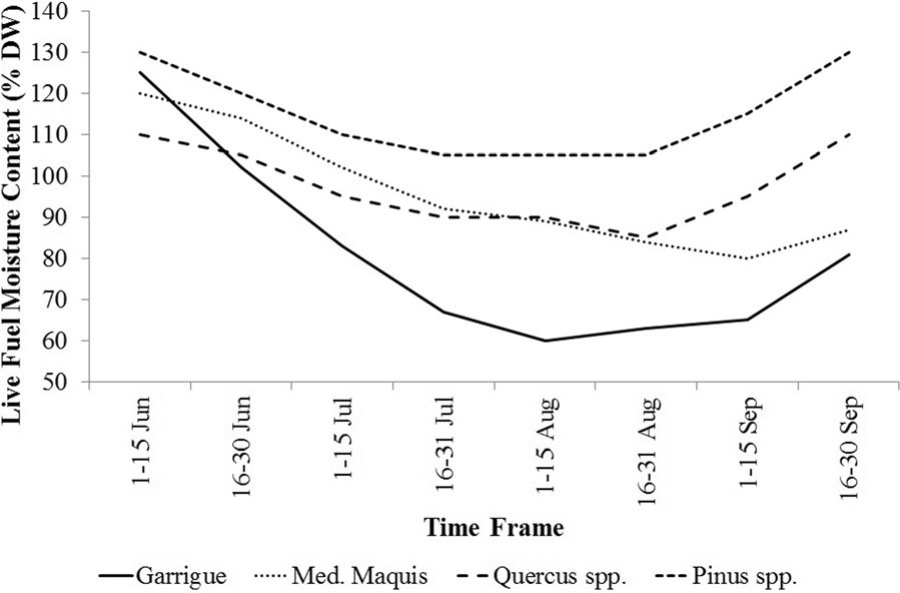
Temporal patterns of average live fuel moisture for different fuel types in Sardinia, from June to September. These data were used as input for the wildfire simulations, as described in the “Methods”

Live and dead fuel moistures for the eight time frames were set using a time series of fuel moisture data collected in northern Sardinia from 2003 to 2011 (Pellizzaro et al. 2007, 2009; personal communication). Live fuel moisture values for garrigue, Mediterranean maquis, *Quercus* spp. and *Pinus* spp. fuel types used in this work are shown in Fig. 5. The seasonal trend of fuel moisture used for the garrigue was determined combining the values measured during field surveys in Sardinia on *Cistus monspeliensis* L., *Helichrysum italicum* (Roth) G. Don, and *Rosmarinus officinalis* L.: These three species represent well the garrigue characteristics in Sardinia. The live moisture average pattern for Mediterranean maquis was determined considering the measurements performed in *Arbutus unedo* L., *Pistacia lentiscus* L., *Phyllirea angustifolia* L., and *Juniperus phoenicea* L. Furthermore, the trends of live fuel moisture of *Pinus* spp. and *Quercus* spp. were determined by combining a set of information and field surveys with data available in literature (Chuvieco et al*.* 2011). The live fuel moisture trend of mixed forests, which in the island are generally combinations of *Quercus* and *Pinus* spp. plantations, was considered equal to *Quercus* spp.

The dead fuel moisture values used in this work (Table 3) resulted from field sampling on litter, dead 1 h, and 10-h fuels. Since dead fuel moisture is variable and strongly affected by weather conditions, specific probabilities of dead fuel moisture conditions were calculated within each time frame. This allowed for weighing the incidence of moderate, low, or extreme dead fuel moistures for each time frame. The data of dead fuel moisture reported in Table 3 were used for all surface fuels.

**Table 3:** Dead fuel moisture parameters used in the fire simulations for each time frame and for each time lag class

	Percentiles of dead fuel moisture values (% DW)					
Time frame/time lag class	*97th*	*90th*	*75th*	*50th*	*25th*	*3th*
1–15 Jun/1 h	9	10	11	13	15	17
1–15 Jun/10 h	13	14	15	17	19	21
1–15 Jun/100 h	15	16	17	19	21	23
16–30 Jun/1 h	8	9	11	12	14	16
16–30 Jun/10 h	12	13	15	16	18	20
16–30 Jun/100 h	14	15	17	18	20	22
1–15 Jul/1 h	7	8	10	11	12	14
1–15 Jul/10 h	11	12	14	15	16	18
1–15 Jul/100 h	13	14	16	17	18	20
16–31 Jul/1 h	7	8	10	11	12	14
16–31 Jul/10 h	11	12	14	15	16	18
16–31 Jul/100 h	13	14	16	17	18	20
1–15 Aug/1 h	8	9	10	11	12	14
1–15 Aug/10 h	12	13	14	15	16	18
1–15 Aug/100 h	14	15	16	17	18	20
16–31 Aug/1 h	9	10	11	12	14	16
16–31 Aug/10 h	13	14	15	16	18	20
16–31 Aug/100 h	15	16	17	18	20	22
1–15 Sep/1 h	9	10	11	12	15	17
1–15 Sep/10 h	13	14	15	16	19	21
1–15 Sep/100 h	15	16	17	18	21	23
16–30 Sep/1 h	10	11	12	13	16	18
16–30 Sep/10 h	14	15	16	17	20	22
16–30 Sep/100 h	16	17	18	19	22	24

The fuel moisture values were defined considering different percentiles and were calculated using data from several years of field sampling campaigns, as described in the “Methods”

#### Wind speed and direction

**Fig. 6:**
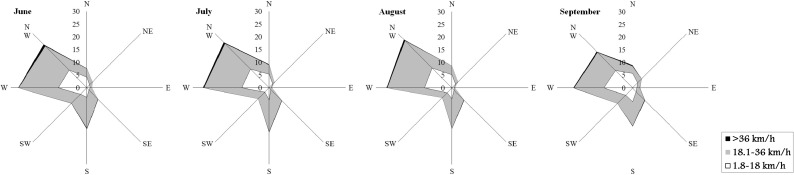
Wind roses for June, July, August, and September in Sardinia. The *axes* report the frequency of each wind direction, in percentage. The weather stations of Alghero (north), Decimomannu (south), and Fonni (center) were used as reference, as described in the “Methods.” Data from Aeronautica Militare Italiana—Servizio Meteorologico (2009)

A set of historical data on wind direction and speed observed in Sardinia during the summer season was gathered from Aeronautica Militare Italiana—Servizio Meteorologico (2009); the probability distribution of wind directions and intensities was calculated at monthly scale combining the wind data of three weather stations (Alghero, Decimomannu, and Fonni) located respectively in the north, south, and center of Sardinia. The data concerning wind speed and direction, together with the associated probability at monthly scale, are shown in Fig. 6.

#### Highly valued resources data

To analyze the spatial and temporal patterns of fire exposure for a set of features of interest during the eight time frames, we selected three highly valued resources (HVRs) of Sardinia: wildland urban interfaces (WUIs), vineyards and orchards (VAOs), and beaches and dunes (BCHs). These features are key values to be protected in case of wildfires, since VAOs are represented by highly valued agricultural areas, and in the surroundings of WUI and BCH human lives, goods and assets are directly threatened by flames and smoke. The HVR data were downloaded in shapefile format from the Sardinia Region website (www.sardegnageoportale.it). For each HVR, a buffer area of 500 m was identified in order to determine wildfire behavior and likelihood in the vicinity of the HVR. To identify the most critical situations in the surroundings of the selected HVR along the time frames, we set burn probability (BP) =0.001 and conditional flame length (CFL) =4.5 m as thresholds of reference. We selected BP =0.001 as reference since it represents a simple indicator for fire management purposes and means that a given pixel will burn once for 1000 fire ignitions. As far as flame length is concerned, we selected the threshold based on the hauling charts of Andrews et al. 2011: flame lengths higher than 4.5 m are indicators of extreme fire behavior conditions (crowning, spotting, and major fire runs), where control efforts at the fire head are ineffective.

### Wildfire simulations

Wildfire simulations were performed by using the minimum travel time (MTT) fire spread algorithm of Finney (2002) as implemented in a command line version of FlamMap called “Randig” (Finney 2006). The MTT algorithm replicates fire growth by Huygens’ principle where the growth and behavior of the fire edge are a vector or wave front (Richards 1990; Finney 2002). The MTT fire spread model was previously calibrated in the study area as reported in several previous studies (i.e., Arca et al. 2012; Salis et al. 2012, 2013). We applied simulation modeling similar to that described in Salis et al. (2013, 2014) to simulate a large number of wildfires and characterize spatial patterns in wildfire likelihood, intensity, and potential size in the island.

In this work, the simulations were stratified into eight time frames, according to the periods described above, and for each time frame, a specific set of fuel moistures and wind fields was used randomly sampling from the historical probabilities of occurrence. Regarding wind fields, the simulations were run using the wind fields produced by the mass-consistent model WindNinja (Forthofer 2007): Specifically, wind fields with definite directions and intensity were created from the historical weather scenarios (Aeronautica Militare Italiana—Servizio Meteorologico (2009)) and then sampled in the simulations for each time frame. Concerning fuel moistures, we used different scenarios with specific likelihood for the different time frames, as described in the previous paragraph and presented in Table 3. The fire ignition points were determined by sampling from a probability grid developed from the historical ignition database for each time frame of the study period (Fig. 3). The ignition probability grid was created with ArcGIS using the inverse distance weighting algorithm (ArcMap Spatial Analyst) and a search distance of 5000 m.

For each time frame, we simulated 50,000 wildfires, sampling wind, weather, and ignitions from period-specific distributions as described above. The fires were simulated for 10-h burn periods of propagation, which represent a common temporal window of active spread for the largest events that affected Sardinia in the last 20 years.

The simulations generated, for each time frame, burn probability maps, and flame length and fire size information for the whole island, at a resolution of 150 m.

The BP represents the likelihood that a pixel will burn considering a single fire ignition in the entire study area and is defined as

1 $$ B{P}_{xy}=\left(\frac{F_{xy}}{N_{xy}}\right) $$

where *F*_*xy*_ is the number of times pixel XY burns and *N*_*xy*_ is the number of simulated fires. Henceforth, BP is a relative measure and is useful for exposure analysis (Ager et al. 2011).

The wildfire intensity depends upon the direction from which the fire encounters a pixel relative to the major direction spread (i.e., heading, flanking, or backing fire), as well as upon slope and aspect (Finney 2002). To estimate fire intensity, Randig converts fireline intensity (FLI, in kW m^−1^) to flame length (FL, in m) based on Byram’s (1959) equation (Wilson 1980):

2 $$ FL=0.0775{(FLI)}^{0.46} $$

Each pixel has a frequency distribution of flame length, which is divided into 20 classes of 0.5-m interval; this distribution, generated from multiple fires burning a pixel, was used to calculate the CFL:

3 $$ \mathrm{C}\mathrm{F}\mathrm{L}={\displaystyle {\sum}_{i=1}^{20}}\left(\frac{{\mathrm{BP}}_{\mathrm{i}}}{\mathrm{BP}\;}\right)\left({F}_i\right) $$

where *FL*_*i*_ is the flame length midpoint of the *i*th category. CFL is the probability-weighted flame length given a fire and is a measure of wildfire hazard (Scott 2006; Ager et al. 2011).

The fire size (FS) output from Randig is the XY ignition coordinate attributed with the area (ha) burned by the fire.

Finally, the temporal variations of BP, CFL, and FS were analyzed by the Kruskal-Wallis test, which is a nonparametric test used for comparing a set of independent samples to determine if the samples come from different populations. Being a nonparametric method, the Kruskal-Wallis test does not assume normal distribution of the residuals. The differences among time steps were tested considering *P* = 0.01.

## Results

### Seasonal patterns of wildfire exposure profiles

**Fig. 7:**
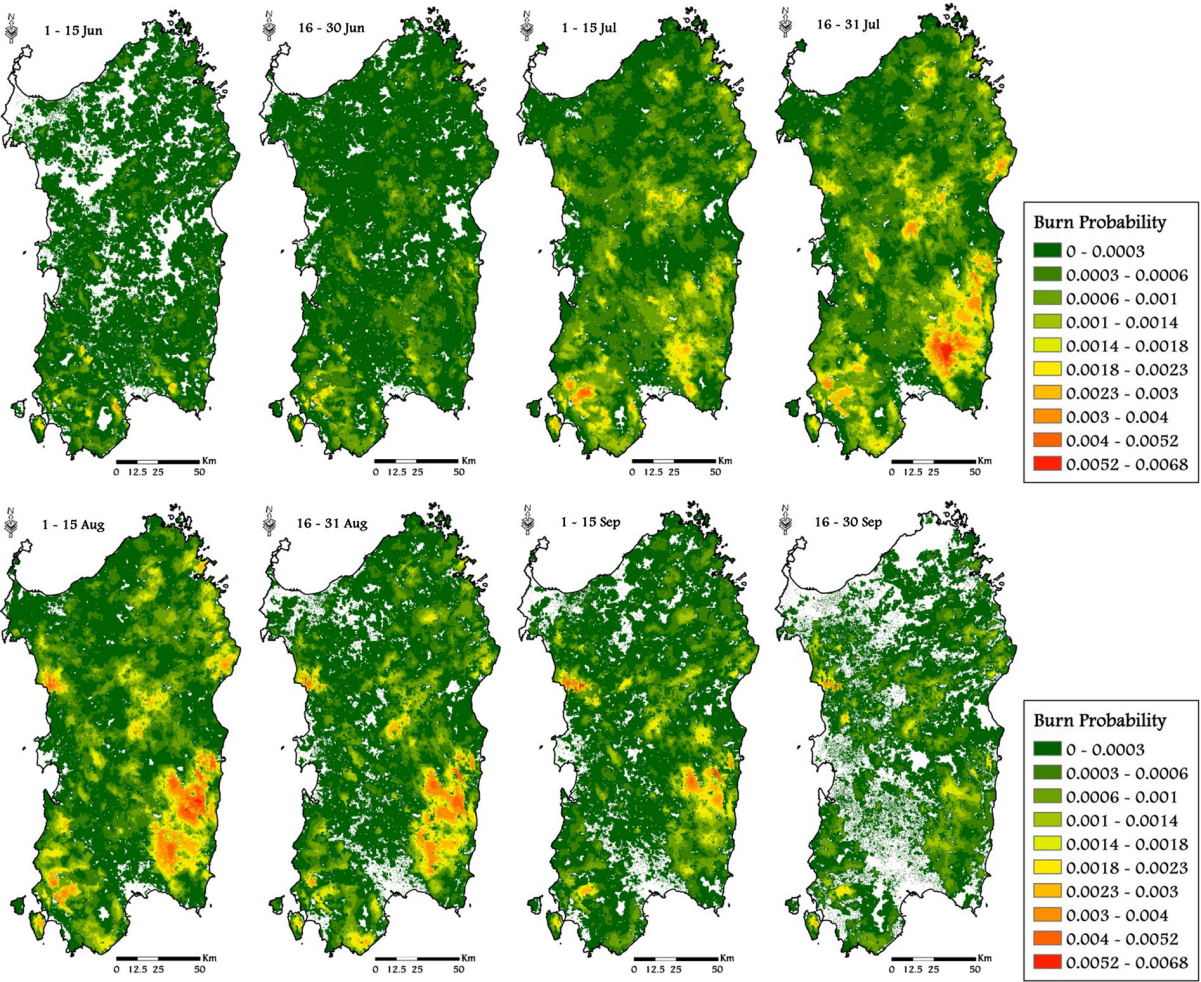
Simulated spatiotemporal patterns of BP in Sardinia, from June to September, considering the historic conditions observed in the study period (1995–2009)

**Fig. 8:**
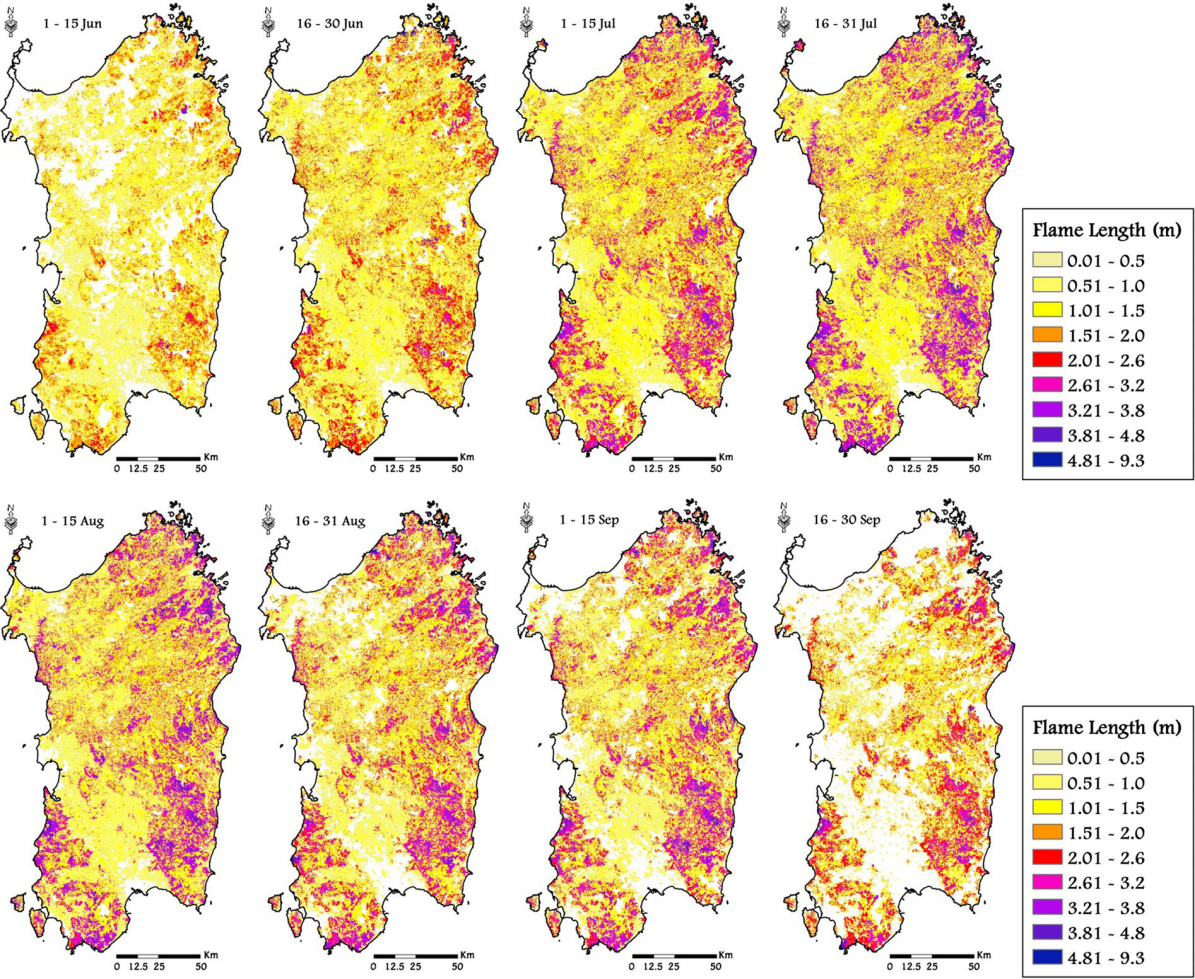
Simulated spatiotemporal patterns of CFL in Sardinia, from June to September, considering the historic conditions observed in the study period (1995–2009)

**Fig. 9:**
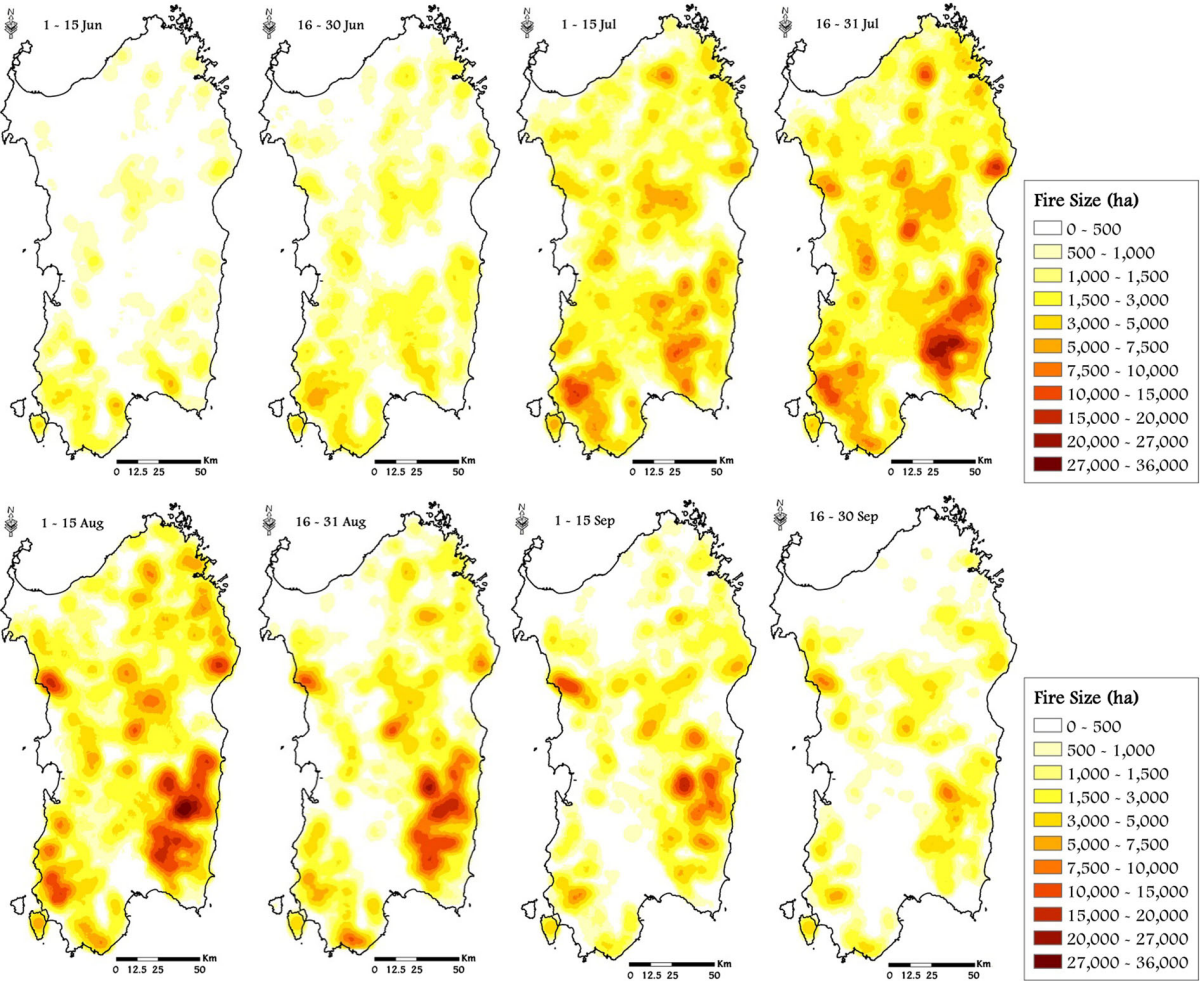
Simulated spatiotemporal patterns of fire size in Sardinia, from June to September, considering the historic conditions observed in the study period (1995–2009)

The Kruskal-Wallis test revealed significant differences (*P* = 0.01, Table 4) among the eight time frames considering all the variables analyzed (BP, CFL, and FS); the differences between time frames were also confirmed using fuel types as classification variable. The seasonal pattern of the mean values of BP, CFL, and FS exhibited a peak in midsummer (time frame 4, 16–31 July), respectively of 6.72 × 10^−4^, 1.53 m, and about 3250 ha (Table 4). The time frame 5 (1–15 August) showed similar but slightly lower values to those of time frame 4. The period with the lowest potential fire exposure was early June, when vegetation has relatively high live and dead moisture content (Table 3 and Fig. 5), thus retarding fire spread. The same conditions occur at the end of September, with strong reductions in BP, CFL, and FS with respect to the previous time frames. Fire intensity, as expressed by conditional flame length, showed similar values between time frames 6 (16-31 August) and 7 (1-15 September) (respectively 1.35 and 1.37 m), in contrast to the patterns for BP and FS. Furthermore, BP, CFL, and FS exhibited a strong spatial variability along the season (Figs. 7, 8, and 9).

**Table 4:** Mean and standard deviation of BP, CFL (m), and FS (ha) for the different time frames, for the whole Sardinia, considering the study period 1995–2009

Time frame	BP	CFL	FS
1–15 Jun	1.50E-04 ± 2.28E-04	0.99 ± 0.57	652.1 ± 663.4
16–30 Jun	2.25E-04 ± 2.53E-04	1.20 ± 0.66	1056.0 ± 890.7
1–15 Jul	4.92E-04 ± 4.68E-04	1.43 ± 0.85	2351.5 ± 1947.4
16–31 Jul	6.72E-04 ± 7.17E-04	1.53 ± 0.97	3256.1 ± 3155.8
1–15 Aug	6.60E-04 ± 8.04E-04	1.47 ± 1.00	3190.3 ± 3622.0
16–31 Aug	4.72E-04 ± 6.77E-04	1.35 ± 0.95	2253.7 ± 2861.5
1–15 Sep	3.66E-04 ± 5.31E-04	1.37 ± 0.99	1727.3 ± 2107.3
16–30 Sep	2.71E-04 ± 3.51E-04	1.21 ± 0.90	1183.3 ± 1202.8

BP, CFL, and FS mean values resulted significantly different for each time frame (Kruskal-Wallis test, *P* < 0.01), as described in the text

The wildfire likelihood was relatively low in time frame 1 (1–15 June), where only small spots in the southern part of the island were characterized by BP values higher than 0.002 (Fig. 7). In time frame 2, also other areas (i.e., southeast, specific areas of the eastern coast and central Sardinia) revealed high values of BP if compared to both the north area and the plains mainly covered by agriculture crops. From 1 to 15 August (time frame 5), the northeastern coastal area and a specific region of central-western Sardinia showed a net increase of BP compared to the previous periods (Fig. 7). After time frame 6, thereafter, a progressive and marked reduction of wildfire likelihood was observed, with low BP values in time frame 8. Yet, in early September, the central-west and south-central-east parts of the island, mainly covered by pastures and grasslands, continued to show relatively high values of BP, due to the agropastoral practices that use fire to renew grazing areas and clean the field from vegetation residuals (Fig. 7).

Fire intensity presented a spatial pattern clearly linked to the fuel types and to the live and dead fuel moisture variations during the season, along with the terrain topography. The peak in fire intensity was observed in time frame 4 (Table 4, Fig. 8), even though from the beginning of July until the end of August, several areas also showed high values of CFL. On the whole, the eastern areas presented values of CFL higher than the rest of the island, probably because of the higher steepness and a greater presence of shrubland and *Quercus* fuel types, in comparison with the large amount of pastures and grasslands of the western sector. The maps showed strong spatial differences between late June and late September (Fig. 8), although the average CFL value was relatively similar (1.20 vs. 1.21 m, Table 4): In time frame 2, in fact, the average CFL was generally low all over the island, while at the end of the fire season, CFL presented greater contrasts and a marked spatial variability (Fig. 8).

In terms of fire size (FS), the maps showed that some areas seemed to support large fire events (Fig. 9). The largest fire sizes were observed in late July. The areas with the most common occurrence of large fires were located in southeast Sardinia and in some spots of central Sardinia. By contrast, large fire events were relatively limited in northern Sardinia (Fig. 9) and at the beginning and the end of the wildfire season, when the largest simulated fires rarely affected more than 5000 ha (Table 4).

The shrubland fuel types (Mediterranean maquis and garrigue) resulted in relevant fire sources since the average BP of these fuel types was the highest among all fuels in several time frames, especially in midsummer (Table 5). The forest types showed a similar trend and evidenced BP values slightly lower than those of the garrigue during the most of time frames. The other fuel types showed much more limited BP, with the lowest values observed for grasslands in September and early June. Overall, Mediterranean maquis presented the highest average CFL, with values above 2.5 m from early July until early September and peaks of about 3.1 m in time frames 4 and 5. The garrigue areas were also characterized by high fire intensity values, with average flame length close or above 1.5 m from early June until mid-September. By contrast, the lowest average CFL was observed in vineyards and orchards, as well as in mixed agricultural areas, both of which are mostly located in flat areas. Regarding forest fuel types, namely conifer and broadleaf stands, and mixed forest, the differences in both BP and CFL were limited, and in some time frames, the variation among conifer and mixed forests was not statistically significant. Generally, broadleaf presented higher values of fire intensity than the other forest types.

**Table 5:** Mean values of BP and CFL (m) for the different time frames, for diverse fuel types in Sardinia, considering the study period 1995–2009

*Time frame*	*Fuel type*	*BP*	*CFL*
1–15 Jun	Grasslands	6.45E-05	0.657
	Mixed agricultural areas	8.05E-05	0.457
	Vineyards and orchards	8.94E-05	0.368
	Herbaceous pastures	1.17E-04	0.556
	Garrigue	1.70E-04	0.903
	Mediterranean maquis	2.41E-04	1.713
	Conifer stands	1.65E-04	0.838
	Broadleaf stands	1.63E-04	1.029
	Mixed forests	2.26E-04	0.981
16–30 Jun	Grasslands	1.48E-04	0.894
	Mixed agricultural areas	1.52E-04	0.653
	Vineyards and orchards	1.55E-04	0.551
	Herbaceous pastures	1.95E-04	0.821
	Garrigue	2.66E-04	1.258
	Mediterranean maquis	3.29E-04	2.114
	Conifer stands	2.39E-04	0.910
	Broadleaf stands	2.31E-04	1.111
	Mixed forests	3.02E-04	1.032
1–15 Jul	Grasslands	3.52E-04	0.964
	Mixed agricultural areas	3.73E-04	0.701
	Vineyards and orchards	3.72E-04	0.611
	Herbaceous pastures	4.71E-04	0.919
	Garrigue	5.90E-04	1.590
	Mediterranean maquis	7.02E-04	2.761
	Conifer stands	4.90E-04	0.988
	Broadleaf stands	4.49E-04	1.215
	Mixed forests	4.99E-04	1.119
16–31 Jul	Grasslands	3.66E-04	0.965
	Mixed agricultural areas	4.34E-04	0.698
	Vineyards and orchards	4.56E-04	0.607
	Herbaceous pastures	6.21E-04	0.899
	Garrigue	8.28E-04	1.722
	Mediterranean maquis	1.05E-03	3.087
	Conifer stands	7.47E-04	1.015
	Broadleaf stands	6.63E-04	1.257
	Mixed forests	7.58E-04	1.162
1–15 Aug	Grasslands	2.71E-04	0.841
	Mixed agricultural areas	3.74E-04	0.625
	Vineyards and orchards	4.16E-04	0.527
	Herbaceous pastures	5.90E-04	0.775
	Garrigue	8.39E-04	1.686
	Mediterranean maquis	1.13E-03	3.067
	Conifer stands	7.54E-04	1.000
	Broadleaf stands	6.55E-04	1.247
	Mixed forests	7.15E-04	1.152
16–31 Aug	Grasslands	9.34E-05	0.690
	Mixed agricultural areas	1.83E-04	0.506
	Vineyards and Orchards	2.36E-04	0.421
	Herbaceous pastures	3.53E-04	0.590
	Garrigue	5.89E-04	1.482
	Mediterranean maquis	8.42E-04	2.760
	Conifer stands	5.80E-04	0.951
	Broadleaf stands	5.18E-04	1.188
	Mixed forests	6.68E-04	1.113
1–15 Sep	Grasslands	9.61E-05	0.678
	Mixed agricultural areas	1.65E-04	0.501
	Vineyards and Orchards	2.05E-04	0.416
	Herbaceous pastures	3.08E-04	0.586
	Garrigue	4.68E-04	1.439
	Mediterranean maquis	6.07E-04	2.823
	Conifer stands	4.30E-04	0.960
	Broadleaf stands	4.00E-04	1.187
	Mixed forests	4.92E-04	1.115
16–30 Sep	Grasslands	5.22E-05	0.324
	Mixed agricultural areas	1.16E-04	0.285
	Vineyards and Orchards	1.49E-04	0.252
	Herbaceous pastures	2.14E-04	0.361
	Garrigue	3.34E-04	1.138
	Mediterranean maquis	4.10E-04	2.331
	Conifer stands	2.89E-04	0.897
	Broadleaf stands	2.62E-04	1.104
	Mixed forests	2.90E-04	1.034

### Seasonal patterns of wildfire exposure to HVRs

**Fig. 10:**
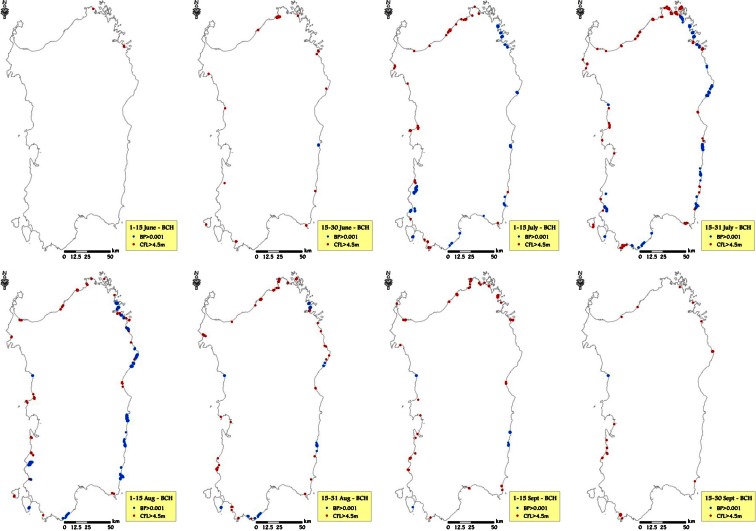
BCH areas characterized by the highest values of burn probability (>0.001) and conditional flame length (>4.5 m) for the different time frames, considering the historic conditions observed in the study period (1995–2009)

Seasonal patterns in exposure were similar for all HVRs, with beaches (BCH) showing the lowest average BP values (Table 6). The peak of wildfire likelihood for beaches was observed in time frame 5 (1–15 August, 2.87 × 10^−4^) and the lowest values at the beginning of June and at the end of September. A different pattern was observed when considering fire intensity, for which the beaches showed the highest values among the HVRs examined after July, with the peak in average CFL close to 1.35 m in time frame 4. As far as the location of the areas most subject to very high wildfire likelihood and intensity is concerned, Fig. 10 shows that several beaches of northeast Sardinia presented values above our thresholds (BP > 0.001 and CFL > 4.5 m), in some cases even at the end of the season. Other areas with relatively high exposure were located in the eastern coast and in southwest Sardinia (Fig. 10).

**Table 6:** Mean and standard deviation of BP and CFL (m) for the different time frames, for selected HVRs in Sardinia, for the study period (1995–2009)

Time frame	BCH		VAO		WUI	
	*BP*	*CFL*	*BP*	*CFL*	*BP*	*CFL*
1–15 Jun	5.49E-05 ± 7.65E-05	0.583 ± 0.508	8.83E-05 ± 1.09E-04	0.551 ± 0.272	9.14E-05 ± 1.39E-04	0.600 ± 0.360
16–30 Jun	9.21E-05 ± 1.32E-04	0.840 ± 0.606	1.77E-04 ± 1.60E-04	0.852 ± 0.251	1.70E-04 ± 1.87E-04	0.932 ± 0.387
1–15 Jul	2.28E-04 ± 2.16E-04	1.204 ± 0.654	4.05E-04 ± 3.15E-04	1.032 ± 0.303	4.18E-04 ± 3.65E-04	1.170 ± 0.447
16–31 Jul	2.85E-04 ± 2.80E-04	1.347 ± 0.791	4.39E-04 ± 4.12E-04	1.068 ± 0334	4.57E-04 ± 4.23E-04	1.230 ± 0.509
1–15 Aug	2.87E-04 ± 3.61E-04	1.208 ± 0.755	3.72E-04 ± 5.11E-04	0.952 ± 0.356	4.18E-04 ± 4.70E-04	1.127 ± 0.527
16–31 Aug	1.41E-04 ± 2.03E-04	1.079 ± 0.805	1.91E-04 ± 3.71E-04	0.731 ± 0.413	2.13E-04 ± 3.19E-04	0.891 ± 0.553
1–15 Sep	1.12E-04 ± 1.67E-04	1.020 ± 0.796	1.68E-04 ± 3.10E-04	0.658 ± 0.410	1.54E-04 ± 2.41E-04	0.825 ± 0.567
16–30 Sep	5.79E-05 ± 1.07E-04	0.506 ± 0.682	8.68E-05 ± 1.57E-04	0.403 ± 0.397	8.31E-05 ± 1.39E-04	0.485 ± 0.519

**Fig. 11:**
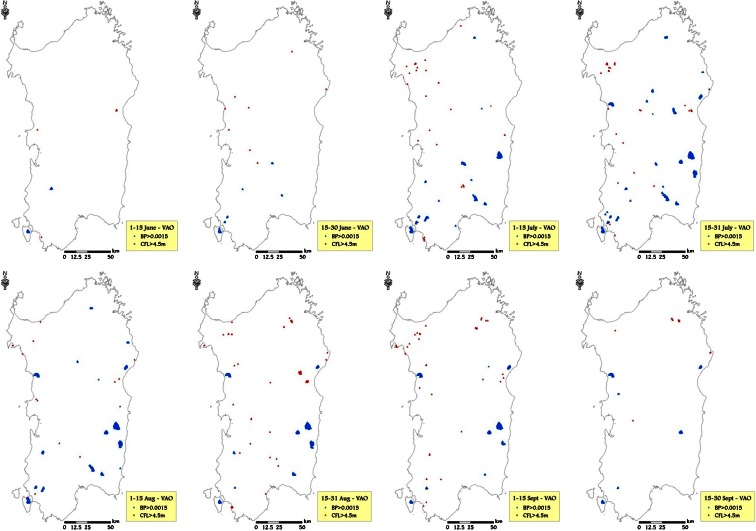
VAO areas characterized by the highest values of burn probability (>0.001) and conditional flame length (>4.5 m) for the different time frames, considering the historic conditions observed in the study period (1995–2009)

Vineyards and orchards (VAOs) had mean BP values very similar to those observed in WUIs, although slightly lower in several cases, and with BP peaks observed in July (respectively 4.05 × 10^−4^ and 4.39 × 10^−4^ in time frames 3 and 4) (Table 6). In terms of CFL, the average fire intensity in VAOs was the lowest among the selected HVRs, with a peak in time frame 4 (1.07 m) and a minimum in time frame 8 (0.40 m). The areas with the highest values of CFL were mainly located in northern Sardinia, while on the other hand, the areas with the greatest wildfire likelihood were concentrated in specific areas of central Sardinia, in particular from early July to early September (Fig. 11).

**Fig. 12:**
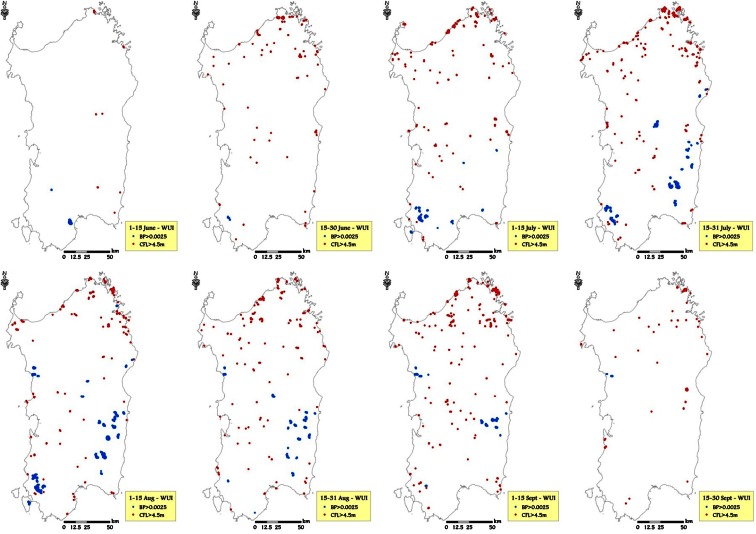
WUI areas characterized by the highest values of burn probability (>0.001) and conditional flame length (>4.5 m) for the different time frames, considering the historic conditions observed in the study period (1995–2009)

Lastly, wildland urban interfaces (WUIs) showed the highest average BP values among the selected HVRs (Table 6). Maximum burn probabilities were observed for fires in the surrounding of WUIs between the beginning of July and early August, with the peak being registered in time frame 4 (4.57 × 10^−4^). The average wildfire intensity in WUI areas was high compared to the other HVRs, especially early in the season (at the beginning and the end of June), and the average CFL from the beginning of July stood at slightly lower values than in BCHs. In time frame 4, the average flame length in WUIs reached a peak of 1.23 m. Regarding the location of areas with the greatest wildfire likelihood and intensity, once again, the northeast coasts of the island were characterized by values above our stated thresholds, early at the end of June and until the end of September. This result indicates that, even from the beginning of the season, some WUIs may be subject to dangerous and severe fire events. In terms of BP, some very well-delimited WUI areas in the south of Sardinia and in the central-western part of the island showed high wildfire likelihood, particularly in July and August (Fig. 12).

## Discussion

This study presented spatiotemporal patterns of wildfire exposure profiles based on spatially explicit burn probability and fire intensity as derived from landscape scale simulation modeling. In particular, this paper addressed the following questions: (1) How does potential wildfire likelihood, size, and intensity differ in the study area at different times during the fire season in Sardinia? (2) How do fuels contribute to the temporal variation in potential wildfire likelihood and intensity? (3) Are highly valued resources and assets characterized by different wildfire exposure profiles during the fire season?

The study area, Sardinia, Italy, is the second largest island of the Mediterranean Basin and had been affected by many severe wildfires over the past 50 years (Boni 2004; Salis et al. 2014). Although in recent years Sardinia experienced a sharp reduction in fire number and area burned, together with the enhancement and strengthening of aerial and terrestrial firefighting forces as well as of their tactics, coordination, and organization, losses and fatalities from wildfires continue, and localized areas and periods of high wildfire risk persist on the island (Salis et al. 2013, 2014). The largest and most severe wildfires of the last years occurred on days with extreme weather and quickly escaped containment efforts of aerial and ground fire suppression resources (Cardil et al. 2013). For these reasons, there is a strong demand from fire agencies, policy makers, and communities to map wildfire risk at fine spatial and temporal scales and design appropriate fuel management and other prevention projects (Piñol et al. 2005, 2007; Calkin et al. 2011a). Wildfire simulation modeling can provide an important contribution to meet these needs when properly calibrated and validated to take into account local conditions (Arca et al. 2007; Duguy et al. 2007; Ager et al. 2011; Calkin et al. 2011a). In our case, the modeling approach provided relevant information in terms of landscape scale spatiotemporal patterns in wildfire exposure on the island that heretofore have not been quantified. The statistical analysis revealed differences among time frames in terms of BP, CFL, and FS that were statistically significant (*P* < 0.01). These differences resulted from combined temporal variation in fuel moisture, fire ignition locations, and winds. Overall, the outputs from the simulation modeling were consistent with historical fire frequency and current knowledge about fire ecology within the study area (Regione Sardegna 2014). Our work identified specific periods in which Sardinia is most susceptible to intense and large fire events (16–31 July and 1–15 August), where the average simulated burn probability of the island was the highest of the fire season, as well as average fire size and flame length. Before and after this period, the fire exposure was observed to be characterized by lower fire likelihood, intensity, and average fire size. The above results are in accordance with other studies conducted over Mediterranean areas (Ganteaume et al. 2012; Ganteaume and Jappiot 2013; Karali et al. 2013), as well as with the official wildfire reports for the island (Regione Sardegna 2014; http://www.sardegnaambiente.it).

We observed clear differences in the ignition spatial distribution of Sardinia, both from coastal toward inner areas, as well as from South to North, particularly early in the fire season, and this spatial pattern influenced the burn probability maps. The spatiotemporal differences in burn probability can be partly explained by the lower potential evaporation and higher local precipitation amounts toward the inner areas (Chessa and Delitala 1997). It is also evident that some BP hot spots at the end of the fire season, mostly located in western Sardinia, result from the agropastoralism practices, which rely on the use of fire to clean the herbaceous and wooded pastures from unwanted vegetation. These areas have shallow rocky soils that make it difficult to use other vegetation control methods such as mechanical cultivation or mowing.

Both weather conditions and phenological variability as exhibited in fuel moisture trends during the fire season, as well as the historical location of ignitions during the different time frames, played a key role in the observed variations in the simulated exposure profiles in the island and affected the differences between southern areas and the rest of Sardinia, particularly early in the season. In terms of topographic effects on fire exposure, we observed the highest burn probability in hilly areas, and moreover, a relationship between modeled fire intensity and slope was evident. Furthermore, intense wildfires were more commonly observed in elevated hills and mountains, where high fuel loads and steep slopes are often combined. Another interesting point we investigated in this work was the influence of fuel models on fire exposure profiles. As expected, shrublands (Mediterranean garrigue and maquis) were characterized by high flame length, as well as high burn probability values, particularly in midsummer. By contrast, the agricultural and herbaceous areas presented on the whole low values of BP and CFL.

The wildfire exposure analysis performed for highly valued resources allowed for examination of exposure profiles of burn probability and fire intensity and illustrated differences in space and time. It is interesting to note that beaches had the highest BP in midsummer, from 16 July to 15 August, which is the period when the tourist pressure is the highest in Sardinia. Furthermore, the average flame length in the surroundings of BCH resulted higher than 1 m from the beginning of July until mid-September. These results suggest that prevention actions in the vicinity of beaches are needed to prevent damaging wildfires, since secure structures for protection are not commonly available in these areas, and natural vegetation is quite often unmanaged. Vineyards and orchards, generally located in managed areas, with low presence of woods and shrubs in the surroundings, presented the average lowest values of CFL, although some areas showed very high BP. The buffer areas of the wildland urban interfaces presented the highest values of BP among all values analyzed, with average BP higher than 4.0 × 10^−4^ in July and early August. In the same period, fire intensity in these areas was on average the highest of the fire season.

On the whole, the maps of burn probability, fire size, and intensity allowed for quantitative representation of wildfire exposure at a scale that is not possible using other approaches such as analysis of historic ignition data, since these latter data do not contain information about fire intensity and do not account for fire spread in the landscape. Work is in progress to couple wildfire simulations with accurate nonparametric statistical models able to account for interactive effects of anthropogenic factors and weather variables on fire occurrence and size, calibrated for the study area, as well as to extend the methods to the whole Italy and other Mediterranean areas (Ager et al. 2014b).

The methodologies we used in this work can inform a spectrum of wildfire management activities, from real-time support during the fire season to fuel management and landscape planning, with the general goal of reducing fire exposure and losses from future wildfires. Moreover, the combination of geo-spatial data with landscape scale fire spread models allows for detailed assessments of the consequences of wildfires burning in different periods or areas (Miller and Ager 2013). Since all municipalities in Sardinia are asked to adopt plans of civil protection and emergency, this work can provide useful guidelines to policy makers and land managers to identify areas at risk and to select the most appropriate prevention and mitigation activities to protect ecosystems and specific values from wildfire losses. Furthermore, this study can help improving landscape scale awareness and understanding of spatial and temporal patterns of wildfire likelihood and intensity, thus allowing to inform different actions aimed to reduce landscape susceptibility and to contrast wildfire spread and ignitions, which in the study area are largely human caused and rarely natural. A detailed knowledge of the timing of wildfires in the different areas of the island can provide sound fundamentals for informing effective decision-support tool and managing fire risk and exposure under climate change scenarios and evolving landscapes. The approach used in this paper can also be used for a range of wildfire effects analyses, including smoke emissions, soil heating, duff consumption, soil erosion, and landslides.
